# Negligible Colon Cancer Risk from Food-Borne Acrylamide Exposure in Male F344 Rats and Nude (*nu/nu*) Mice-Bearing Human Colon Tumor Xenografts

**DOI:** 10.1371/journal.pone.0073916

**Published:** 2013-09-05

**Authors:** Jayadev Raju, Jennifer Roberts, Chandni Sondagar, Kamla Kapal, Syed A. Aziz, Don Caldwell, Rekha Mehta

**Affiliations:** 1 Toxicology Research Division, Bureau of Chemical Safety Food Directorate, Health Products and Food Branch, Health Canada, Ottawa, Ontario, Canada; 2 Scientific Services Division, Bureau of Chemical Safety Food Directorate, Health Products and Food Branch, Health Canada, Ottawa, Ontario, Canada; Rush University Medical Center, United States of America

## Abstract

Acrylamide, a possible human carcinogen, is formed in certain carbohydrate-rich foods processed at high temperature. We evaluated if dietary acrylamide, at doses (0.5, 1.0 or 2.0 mg/kg diet) reflecting upper levels found in human foods, modulated colon tumorigenesis in two rodent models. Male F344 rats were randomized to receive diets without (control) or with acrylamide. 2-weeks later, rats in each group received two weekly subcutaneous injections of either azoxymethane (AOM) or saline, and were killed 20 weeks post-injections; colons were assessed for tumors. Male athymic nude (*nu/nu*) mice bearing HT-29 human colon adenocarcinoma cells-derived tumor xenografts received diets without (control) or with acrylamide; tumor growth was monitored and mice were killed 4 weeks later. In the F344 rat study, no tumors were found in the colons of the saline-injected rats. However, the colon tumor incidence was 54.2% and 66.7% in the control and the 2 mg/kg acrylamide-treated AOM-injected groups, respectively. While tumor multiplicity was similar across all diet groups, tumor size and burden were higher in the 2 mg/kg acrylamide group compared to the AOM control. These results suggest that acrylamide by itself is not a “complete carcinogen”, but acts as a “co-carcinogen” by exacerbating the effects of AOM. The nude mouse study indicated no differences in the growth of human colon tumor xenografts between acrylamide-treated and control mice, suggesting that acrylamide does not aid in the progression of established tumors. Hence, food-borne acrylamide at levels comparable to those found in human foods is neither an independent carcinogen nor a tumor promoter in the colon. However, our results characterize a potential hazard of acrylamide as a colon co-carcinogen in association with known and possibly other environmental tumor initiators/promoters.

## Introduction

The discovery of acrylamide, a Class 2A probable human carcinogen [1] and rodent carcinogen [2–5], in popular and fast foods [6] has raised public health concerns [7]. High levels of acrylamide are formed in foods processed at high temperatures (baking and frying) through the Maillard reaction between the amino group of asparagine and the carbonyl group of reducing sugars [8]. Foods such as French fries, potato and tortilla chips, baked foods, breakfast cereals and roasted coffee contain acrylamide at parts per million concentrations [9–13]. A recent study found that non-breastfed infants aged 6–12 months were exposed to acrylamide from commercial foods with the mean acrylamide level determined to be 73 µg/kg in formula powder product and 10.5 µg/L in drinkable product [14]. Once ingested by humans, acrylamide and its epoxide metabolite glycidamide can bind to hemoglobin and DNA to form adducts, and can interact with other proteins at the cellular level [15]. The toxicokinetics, dose response and hazard identification of acrylamide have been elegantly reviewed by Shipp et al. (2006) [15]. The literature is replete with experimental data supporting the risk characterization of acrylamide as a carcinogen, genotoxin and neurotoxin.

In a 2-year rodent study conducted according to OECD Test Guideline-451, acrylamide (administered in the drinking water) increased the tumor incidence within the thyroid gland and oral cavity in both sexes, as well as the breast gland in females, and testes in males [4]. Studies in various mouse models of skin and lung cancer demonstrated that acrylamide could act both independently as a tumor initiator or depended on a promoting agent for its carcinogenic activity, depending on the choice of animal model and route of administration [2,3]. In a life-time rodent study, acrylamide (in drinking water) increased the tumor incidence within the breast gland in females and testicular gland in males [5]. In a study in Syrian hamsters, acrylamide-induced toxicity (administered through drinking water) caused peripheral nerve disorders, hematotoxicity and testicular toxicity [16]. The 2-year cancer bioassay [4] and the life-time carcinogenicity study [5] have provided seminal data describing the carcinogenicity of acrylamide, and colon was not identified as a target organ for acrylamide’s action. The recently published in-depth toxicological evaluation of acrylamide by the National Toxicology Program of the United States Department of Health and Human Services confirmed these findings [17]. In their report, the stomach of male and female B6C3F1 mice was identified as an additional target of acrylamide in the 2-year carcinogenicity studies (acrylamide administered in the drinking water at 0.0875, 0.175, 0.35 or 0.70 mM/kg body weight), with clear evidence of a dose-related incidence of forestomach squamous cell papilloma or carcinoma [17]. Findings from two epidemiological studies suggested that no association exists between dietary acrylamide and colon cancer risk in women [18] or men [19]. Contrary to these epidemiological and other experimental evidences, Zhang (2009) reported on a study conducted with male Sprague-Dawley rats, addressing the induction of colon aberrant crypt foci (ACF, putative colon precancerous lesions) and tumor formation by i.p. exposure to acrylamide at a dose of 10 mg/kg body weight administered 5 days a week over a period of 8 weeks [20]. It was reported that the number of acrylamide-induced colon ACF and tumors were significantly higher in rats on the 10% corn oil-supplemented diets compared to those on the basal diet [20]. In another study, rats fed diets supplemented with 10% olive oil and 10% fish oil showed decreased acrylamide-induced ACF formation in comparison to those on the basal diets [21]. These two studies implicate acrylamide as an independent colon carcinogen, especially in the absence of any known carcinogen used in their protocol [20,21]. To address whether dietary acrylamide at doses known to cause multiple tumors in rodents would increase the risk of colon cancer, we previously conducted a short-term bioassay (8-week study design) using the azoxymethane (AOM)-induced rodent colon ACF as a surrogate biomarker [22]. We reported that dietary acrylamide (5 to 50 mg/kg diet) did not increase ACF formation by comparison to the control (no acrylamide in the diet) in either low- or high-fat diet regimens; however, a trend to increase ACF was observed at the lowest tested dose (5 mg acrylamide per kg diet) [22]. The exact modes of action of acrylamide in the multistep carcinogenic process, as a tumor initiator, promoter or co-carcinogen, remain unclear from these studies. In addition, the scope of the effect of low and chronic oral acrylamide exposure, especially at levels reflecting those found in human foods, is unclear. The doses of acrylamide used in most animal studies are significantly higher than those to which humans are exposed through the diet. Thus, the main objective of the present study was to assess if acrylamide in the diet, at doses reflecting the higher human exposure levels, causes any carcinogenicity in the colon, using two established animal models. Dietary acrylamide did not independently initiate colon tumor formation in F344 rats; however, when administered along with AOM, a colon specific carcinogen, acrylamide (2 mg/kg diet) increased the size of colon tumors suggesting a co-carcinogenic effect in the colon. Additionally, dietary acrylamide did not exacerbate the growth of human colon tumor xenografts in nude (*nu/nu*) mice.

## Methods

### Ethics statement

The animal experimental protocol of this study was approved by the Health Canada Ottawa Animal Care Committee (ACC No. 2010-015), and animals were cared for according to the Guidelines of the Canadian Council for Animal Care.

### Animals, husbandry and diets

**Figure 1:**
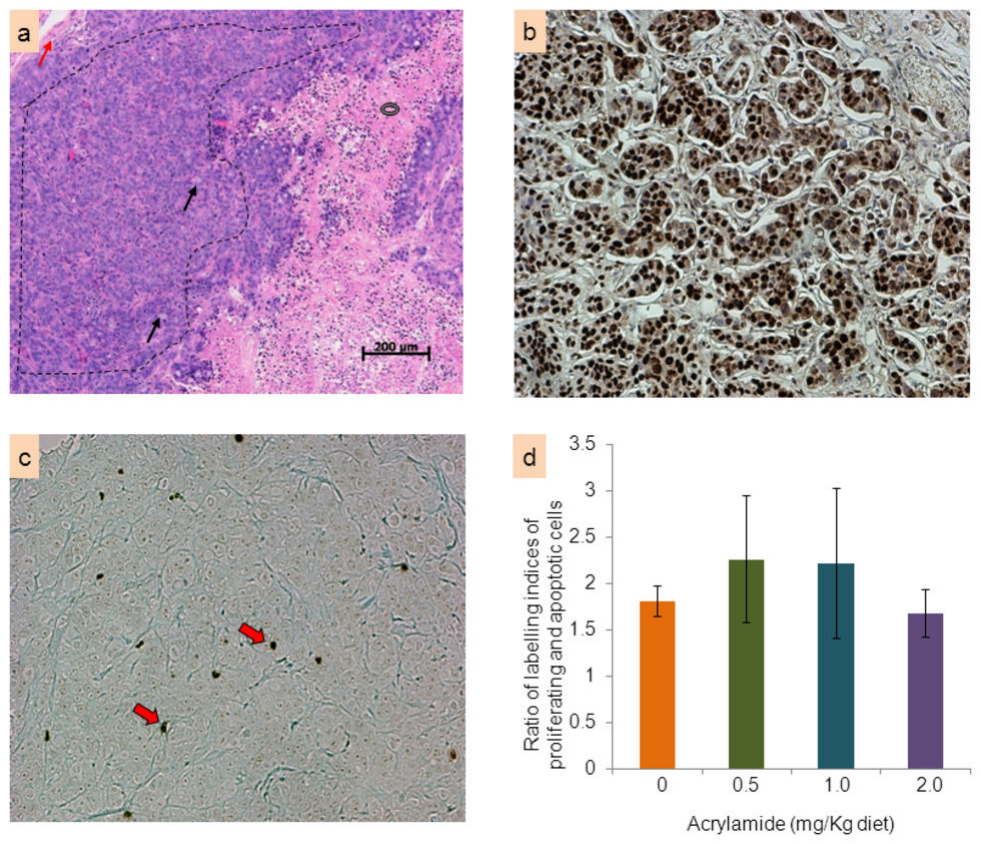
Experimental design of (a) the F344 rat study and (b) the nude (*nu/nu*) mouse- human colon tumor xenograft study. Numbers in boxes depict the time (in weeks) after the animals arrived at our housing facility. In both studies, a one-week acclimatization period was maintained before specific interventions. Diets were based on AIN-93G semi-synthetic formulation and the four experimental diets differed from one another in the level of acrylamide added: 0 (control), 0.5, 1.0 and 2.0 mg/kg diet. All animals remained on the respective experimental diets until necropsy.

Male F344 rats and nude (*nu/nu*) mice, weaned on to a semi-synthetic diet [23], were procured from Charles River Laboratories (Quebec, Canada). On arrival to our animal housing facility, all animals were acclimatized for 1 week under laboratory conditions. Temperature and relative humidity were controlled at 22°C and 55%, respectively, with a 12 h light/12 h dark cycle. Animals had free access to experimental diets and drinking water *ad libitum*. Diets were obtained from Research Diets, Inc. (New Brunswick, New Jersey, USA) in the form of powder and were based on AIN-93G semi-synthetic formula modified to contain 7% corn oil [23]. Acrylamide (≥ 99% pure; Sigma Chemical Co., Missouri, USA) at doses of 0.5 mg, 1.0 mg and 2.0 mg per kg diet was mixed with the diets using a Hobart mixer and then made into pellets using a pelleting press (temperature never exceeded 35°C) in our diet preparation facility. The diet without any addition of acrylamide (0 mg) represented the basal diet. Diets were stored in the dark at 4°C until use. Diets were replenished and food consumption was measured weekly. Rats were monitored every day and their body weights were recorded twice a week. Animals were killed by exsanguination under isoflurane anaesthesia prior to collecting tumors and organs. The specific experimental design for the two studies using F344 rats and nude mice are shown in Figure 1.

### Experimental design and tumor calculations

#### F344 rat study

Male F344 rats (7 weeks of age) were randomized to one of the four experimental diets. After 2 weeks, rats within each diet group were sub-divided to receive s.c. injections of either AOM (Sigma Chemical Co., Missouri, USA; 15 mg/kg BW; n = 24 rats/diet group) or saline (0.2 ml/rat; n = 8 rats/diet group) once a week for 2 weeks (see Figure 1). All rats were on the experimental diets for 20 weeks post AOM/saline injections, after which they were killed. Colons were dissected, flushed with ice-cold PBS, and slit open along the length from the anus to the cecum on a cold plate. Macroscopic lesions and tumors were assessed for size and location along the colon, were dissected and snap-frozen in RNA*later*™ stabilizing agent (Life Technologies, Grand Island, New York, USA) in liquid N_2_ for molecular analysis or stored in 10% buffered formalin for pathology. Segments (1 cm) of the distal and proximal sections of the colons were dissected out and fixed flat between filter papers in 10% buffered formalin for pathology; the rest of the colons (distal and proximal separately) were scraped with glass slides to collect the mucosal layer and snap-frozen in liquid N_2_ for future analysis. Colon tumor parameters derived from the tumor size and location data recorded at the time of necropsy include: (a) tumor incidence (percentage of total animals with tumors); (b) tumor multiplicity (mean number of tumors per tumor-bearing rat); (c) mean tumor size (mm^2^) per group; and (d) tumor burden (mean of total tumor area per tumor-bearing rat) as described by 24.

#### Nude (nu/nu) mouse study

Male athymic nude (*nu/nu*) mice (6 weeks of age) were housed in a Level-II isolation facility and maintained under sterile conditions. A week after acclimatization, mice were injected subcutaneously in the right shank with HT-29 human colon adenocarcinoma cells (2 × 10^6^ cells) suspended in 100 µl serum-free McCoy’s 5A media (Hyclone Laboratories Inc., South Logan, Utah, USA). After 3 weeks, the mice were randomized to one of the 4 experimental diets (n = 12 mice/diet group) (see Figure 1). Mice were palpated twice a week and tumor xenografts measurements were recorded independently by two technicians. Four weeks later, mice were killed; the tumor xenografts were weighed and either stored in 10% buffered formalin for pathology together with un-involved surrounding tissue or snap-frozen in liquid N_2_ for molecular analysis. Tumor measurements were recorded for 5 time periods, beginning from the week the acrylamide diets were introduced until necropsy. Tumor volume was calculated using the formula 0.5236 × L_1_(L_2_)^2^, where L_1_ and L_2_ are the diameters of each tumor at the longest and shortest points, respectively. Tumor growth was calculated as the change in tumor volume per week (represented as percent) beginning at the introduction of the experimental diets (3 weeks post injection with cells) for a total of 4 weeks.

### Tumor pathology

Formalin fixed colon tumors (together with adjacent normal-appearing mucosa) from the AOM-injected F344 rats and the tumor xenografts from nude mice were embedded in paraffin, sectioned at 5 µm, and stained with hematoxylin and eosin (H&E) for pathological classification. Colon tumors from the F344 rats were classified according to Whiteley et al. (1996) [25], and the colon tumor xenografts from nude mice were classified according to the WHO scheme for tumors of the colon and rectum [26]. In the nude mouse study, in addition to the xenograft tumors from each mouse, other organs (brain, liver, lung, and the inguinal lymph node) were collected and examined microscopically for metastases.

### Immunohistochemical quantification of proliferation and apoptosis

Unstained sections (5 µm thick) of the colon xenografts from the nude mice were used for all immunohistochemical detections. For proliferation, sections were first subjected to antigen retrieval by heating in 10 mmol/L sodium citrate buffer in a microwave for 2.5 minutes. The mouse monoclonal anti-proliferating cell nuclear antigen (PCNA) (Cat. # M0879; DakoCytomation, Carpinteria, CA, USA) at a dilution of 1:10000 was used as the primary antibody. The EnVision™+ System (Cat. # K4007, DakoCytomation, Carpinteria, CA, USA), using an anti-mouse secondary antibody conjugated with horseradish peroxidase and subsequent detection with diaminobenzidine, was used according to the manufacturer’s instructions. For apoptosis, the ApopTag® Plus Peroxidase In Situ Apoptosis Kit (Cat. # S7101; Chemicon Inc., Canada) was used according to the manufacturer’s instructions. This terminal deoxynucleotidyl transferase dUTP nick end labeling (TUNEL) assay-based method detects early apoptosis via DNA fragmentation by enzymatically labeling the free 3'-OH termini with modified nucleotides. Briefly, sections were subjected to a protein digestion enzyme to quench endogenous peroxidase, followed by application of an equilibration buffer and incubating with TdT enzyme, before stopping the reaction by adding anti-digoxigenin conjugate buffer. This was followed by incubating the sections in the peroxidase substrate and counterstaining using methyl green.

For determining the labeling index of mitotic cells (PCNA-positive) and apoptotic cells (TUNEL-positive), ten well-oriented fields were evaluated per colon xenograft section. The PCNA/apoptosis labeling index was calculated using the software Northern Eclipse Version 7.0 (Empix Imaging, Inc., Mississauga, ON, Canada). A ratio of mitotic/apoptotic cells per section was calculated.

### Statistical analysis

Data were analyzed by one-way ANOVA using SigmaStat® 3.1 software (Systat Software Inc., Point Richmond, California, USA). In all statistical tests, *p* < 0.05 was considered significant.

## Results

### F344 rat study

#### General observations

**Figure 2:**
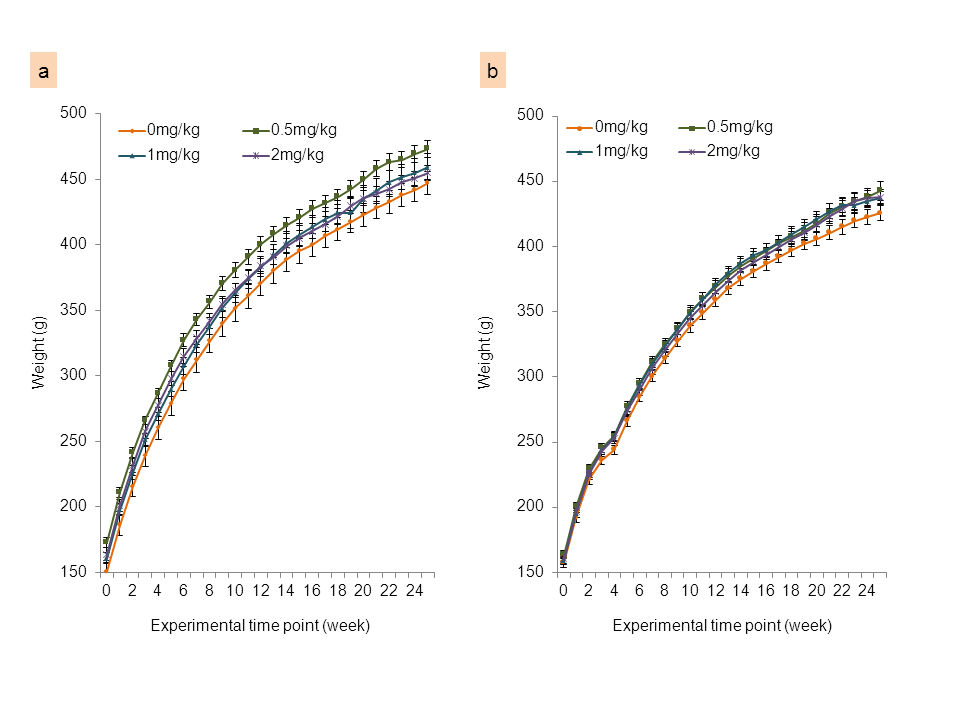
Body weight gain of F344 rats. Body weight (mean ± SE, g) per week of F344 rats injected with (a) saline or (b) AOM and treated with acrylamide at 0 (control), 0.5, 1.0 and 2.0 mg/kg diet.

Body weight (mean ± SEM) of rats at the beginning of the experiment (at the time of the first injection) was 228.8 ± 5.5 g and 225.7 ± 3.6 g for saline and AOM groups, respectively. No significant differences in body weight gain between control (0 mg acrylamide) and acrylamide-treated rats were found in either saline or AOM groups (Figure 2). Final body weights of rats did not differ significantly among the different diet groups in saline- or AOM-injected rats (Table 1). No differences were observed for liver and spleen weights (calculated as g per kg body weight) between the acrylamide-treated groups and their respective controls in either saline- or AOM-treated groups (Table 1). Food consumption was calculated as mean values (g per kg body weight per day), and was not different between control and acrylamide-treated rats in either saline or AOM groups (Table 1). Based on the recorded food consumption for each individual dietary group in the saline-treated rats, the intake of acrylamide for concentrations of 0.5, 1 and 2 mg acrylamide per kg diet used in our study were calculated as 0.017, 0.035 and 0.070 mg acrylamide per kg body weight per day, respectively. A similar trend was observed in the AOM-treated rats, with the calculated intake of 0.018, 0.036 and 0.072 mg acrylamide per kg body weight per day for concentrations of 0.5, 1 and 2 mg acrylamide per kg diet, respectively.

**Table 1:** Body and organs weights of saline- and AOM-injected rats fed diets with or without acrylamide for 24 weeks.^a,b^

	Control	Acrylamide		
		0.5 mg/kg	1.0 mg/kg	2.0 mg/kg
Saline-injected rats (*n* = 8/group)				
Body Weight	459.5 ± 7.9	483.9 ± 7.4	469.5 ± 9.8	465.6 ± 5.9
(g)				
Liver	3.14 ± 0.04	3.19 ± 0.05	3.13 ± 0.03	3.08 ± 0.06
(g/100g BW)				
Spleen	0.173 ± 0.002	0.182 ± 0.004	0.180 ± 0.003	0.181 ± 0.002
(g/100g BW)				
Food intake	35.4 ± 0.2	34.8 ± 0.1	34.7 ± 0.2	35.2 ± 0.1
(g/kg BW/day)				
AOM-injected rats(*n*= 24/group)				
Body Weight	424.4 ± 5.7	440.3 ± 7.5	436.0 ± 5.9	436.2 ± 5.9
(g)				
Liver	2.79 ± 0.03	2.80 ± 0.06	2.77 ± 0.05	2.75 ± 0.04
(g/100g BW)				
Spleen	0.216 ± 0.023	0.215 ± 0.023	0.187 ± 0.003	0.189 ± 0.003
(g/100g BW)				
Food intake	36.3 ± 0.5	35.8 ± 0.4	35.9 ± 0.4	35.5 ± 0.4
(g/kg BW/day)				

^a^ All values recorded at the time of necropsy, 20 weeks post saline/AOM injections; ^b^ mean ± SE; and; ^*^ statistically significant from control (0 mg/Kg acrylamide/diet) at *p* < 0.05.

Regardless of the dietary treatment, none of the rats exhibited signs of toxicity, discomfort or behavioural anomalies until 18 weeks post-AOM/saline injection. However, at 19 weeks post-AOM/saline injections, more than 30% of the AOM-injected rats had blood in their stool while none of the saline-injected rats had such signs. When comparing this effect (percent of rats with blood in their stool/group) across dietary groups, the only notable observation was that more AOM-injected rats at the highest dose of acrylamide (2 mg/kg diet) had blood in their stool (41.7%) compared with the control (29.2%) and the other two dose groups of 0.5 and 1 mg/kg (25.0% and 29.2%, respectively). The study was therefore terminated at 20 weeks, 6 weeks ahead of the originally proposed time.

#### Tumor data

At the end of 20 weeks post-AOM/saline injection, none of the saline-injected rats on either the control or acrylamide diets had developed colon tumors (Table 2). A colon tumor incidence of 54.17% was observed in the AOM-injected rats on the control diet. No significant differences were observed in the tumor incidences between control and the acrylamide-treated groups (*p* = 0.695). Tumor multiplicity was similar among the different dietary groups. However, the average size of tumors per rat and the tumor burden (total tumor area per tumor-bearing rat) was significantly higher (*p* < 0.05) in the highest acrylamide dose group of 2 mg/kg diet compared to the control. No difference in tumor size and burden was noted in the other two acrylamide groups of 0.5 mg/kg diet (*p* = 0.933, *p* = 0.959, respectively) and 1 mg/kg diet (*p* = 0.978, *p* = 0.741, respectively).

**Table 2:** Colon tumor parameters of saline- and AOM-injected rats fed diets with or without acrylamide for 24 weeks.^a^

		Control		Acrylamide			
				0.5 mg/kg	1.0 mg/kg		2.0 mg/kg
Saline-injected rats							
Total rats		8		8	8		8
Tumor-bearing rats		0		0	0		0
Tumor incidence^b^		0		0	0		0
AOM-injected rats							
Total rats		24		24	24		24
Tumor-bearing rats		13		14	12		16
Tumor incidence^b^		54.17		58.33	50.00		66.67
Total Tumors		22		19	16		25
Tumor multiplicity^c^		1.69 ± 0.32		1.36 ± 0.18	1.33 ± 0.14		1.56 ± 0.24
Tumor size^c^ (mm^2^)		10.68 ± 1.85		6.91 ± 1.16	13.68 ± 4.04		25.18± 8.34*
Tumor burden^c,d^ (mm^2^)		19.27 ± 3.92		8.75 ± 2.74	17.93 ± 3.11		35.52 ± 9.96*

^a^ All values recorded at the time of necropsy, 20 weeks post saline/AOM injections; ^b^ % rats with tumors; ^c^ mean ± SE; ^d^ total area occupied by tumors in each tumor-bearing rat in the group per total number of tumor-bearing rats in the group; and ^*^ statistical different from control (0 mg/kg acrylamide/diet) at *p* < 0.05.

**Figure 3:**
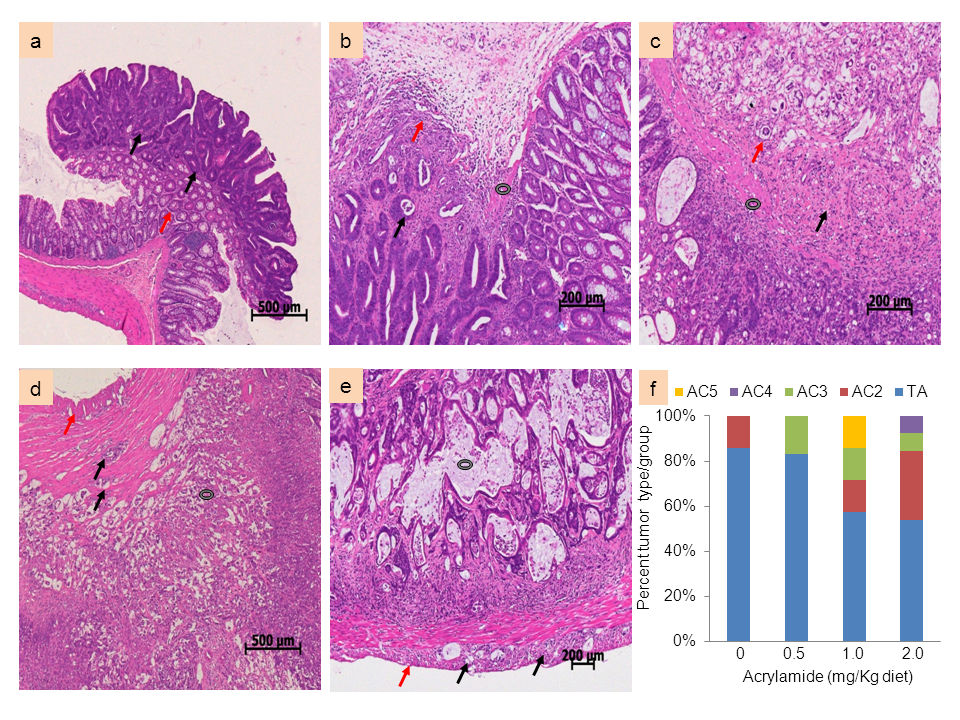
Histopathological identification of lesions (5 µm thick, H&E-stained sections) in the colon of AOM-injected F344 rats. One of the five tumor types were observed in the colons. Tubular adenoma (TA) is shown in panel (a), with dysplastic tubules forming the polypoid adenoma (black arrow) and non-neoplastic glands in the stalk of the tumor (red arrow). Adenocarcinoma with invasion to submucosa (AC-2) is shown in panel (b), featuring neoplastic glandular structures (black arrow) in submucosa with a clear muscularis mucosa (black donut) and scirrhous inflammatory response to tumor (red arrow). Mucinous adenocarcinoma with invasion to submucosa (AC-3) is shown in panel (c), characterised by tumor cells forming glandular structures (red arrow) in submucosa with a clear muscularis mucosa (black donut) and signet-ring cells distended by mucin (black arrow). Mucinous adenocarcinoma invading tunica muscularis (AC-4) is shown in panel (d), characterised by tumor cells invading tunica muscularis (black arrow) and replacing normal mucosa (black donut) and an intact serosa (red arrow). Mucinous adenocarcinoma invading serosa (AC-5) is shown in panel (e), with tumor cells in vascular structures in tunica muscularis (black arrow), mucous and debris in dilated tumor gland (black donut), and a serosa (red arrow). Tumor type as a percentage in each diet group is depicted in panel (f).

Within the classification framework suggested by Whiteley et al. (1996) [25], colon tumors in the AOM-injected rats fell in the following categories: (a) tubular adenoma (TA), in which at least 75% of the mass consists of well differentiated tubules; (b) adenocarcinoma (AC)-2 invading into submucosa; (c) AC-3 representing mucinous-type lesion invading into submucosa; (d) AC-4 representing mucinous lesion invading into tunica muscularis; and (e) AC-5 representing mucinous lesion invading to serosa (Figure 3). In the control (AOM alone, no acrylamide) group, 80% of the lesions were adenomas (TA type) and 20% were adenocarcinomas (AC-2 type). A similar trend was observed in the lowest acrylamide group (0.5 mg/kg diet) except that the adenocarcinomas were of the mucinous type (AC-3) (Figure 3). Only 55% and 44% adenomas were found in the medium (1 mg/kg diet) and high (2 mg/kg diet) acrylamide groups, respectively, the remainder falling under adenocarcinomas with more advanced mucinous characteristics (AC-4 and AC-5) (Figure 3).

In addition to colon tumors, we observed tumors of the small intestine (duodenum, jejunum or ileum) in the AOM-injected rats with an incidence of 12.5% in the control group, and 20.8%, 25.0% and 8.3% in the 0.5 mg/kg, 1.0 mg/kg and 2.0 mg/kg acrylamide/diet groups, respectively.

### Nude (nu/nu) mouse study

#### General observations

**Figure 4:**
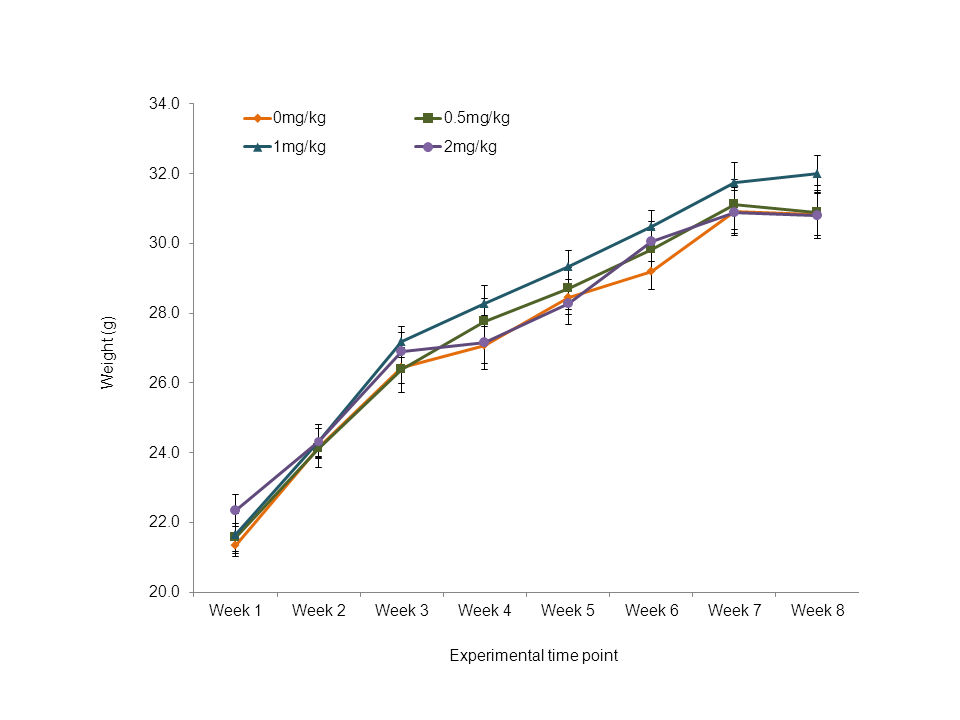
Body weight gain of nude (*nu/nu*) mice. Body weights (mean ± SE, g) of nude (*nu/nu*) mice injected with HT-29 human colon cancer cells treated with acrylamide at 0 (control), 0.5, 1.0 and 2.0 mg/kg diet.

No differences in body weight gain between control (0 mg/kg acrylamide/diet) and the three acrylamide groups were found (Figure 4). Mice appeared healthy with no visible signs of toxicity, discomfort or behavioral anomalies during both the tumor-formation phase (first 3 weeks following injection of the cancer cells) and the acrylamide-feeding phase. The food consumption was not different between control and acrylamide-treated mice, and irrespective of dietary treatments was recorded as 12.30 ± 0.52 g/kg BW/day. The intake of acrylamide for doses of 0.5, 1 and 2 mg acrylamide per kg diet used in the mice study were calculated as 0.006, 0.012 and 0.025 mg acrylamide per kg body weight per day, respectively.

#### Tumor xenograft data

Three weeks after HT-29 human colon adenocarcinoma cells were injected in the right shank region of nude mice, subcutaneous masses appeared at the injection sites in all but 4 mice. The masses were discrete, firm, palpable, and felt localized to the subcutaneous region. The 4 mice that did not have palpable tumor xenografts were excluded from the study. The rest of the mice received either the control or one of the three experimental diets (0.5, 1.0 and 2.0 mg acrylamide/kg diet) for a total of 4 weeks.

**Figure 5:**
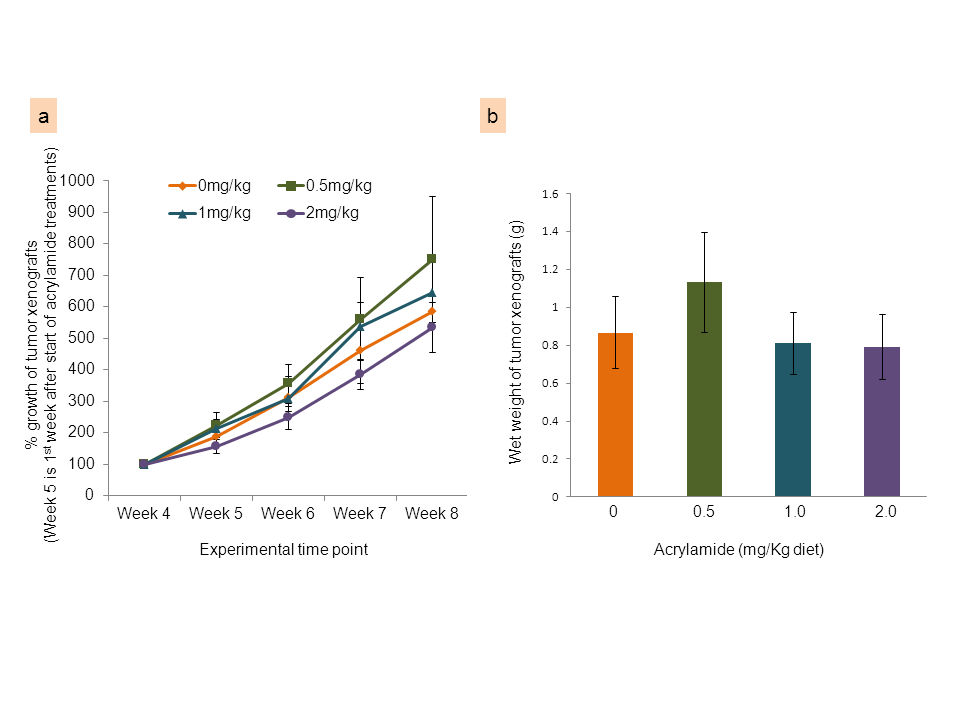
Tumor xenograft characteristics in nude (*nu/nu*) mice. Effect of dietary acrylamide at 0 (control), 0.5, 1.0 and 2.0 mg/kg diet on growth (mean ± SE, %) shown in panel (a) and wet weights (mean ± SE, g) shown in panel (b) of HT-29 human colon tumor xenografts in nude (*nu/nu*) mice. Tumor growth was calculated based on the volume of each tumor recorded at different time points from the beginning of the acrylamide diets until necropsy, and wet weights were recorded at necropsy.

At termination, all tumor xenografts appeared to have intact skin except for two mice (one each in the 0.5 and 1.0 mg acrylamide/kg diet groups) that had focal ulcerations in the tumor xenografts. One mouse from the 1.0 mg acrylamide/kg diet group had an enlarged lymph node (inguinal), with no metastasis when observed histologically. There were no differences in tumor growth between the control and acrylamide-treated groups at any of the 4 time points (beginning one week after initiating the acrylamide diet), including at necropsy (Figure 5). The wet weight of the tumors at necropsy was similar across diet groups, with no acrylamide-related change (Figure 5).

**Figure 6:**
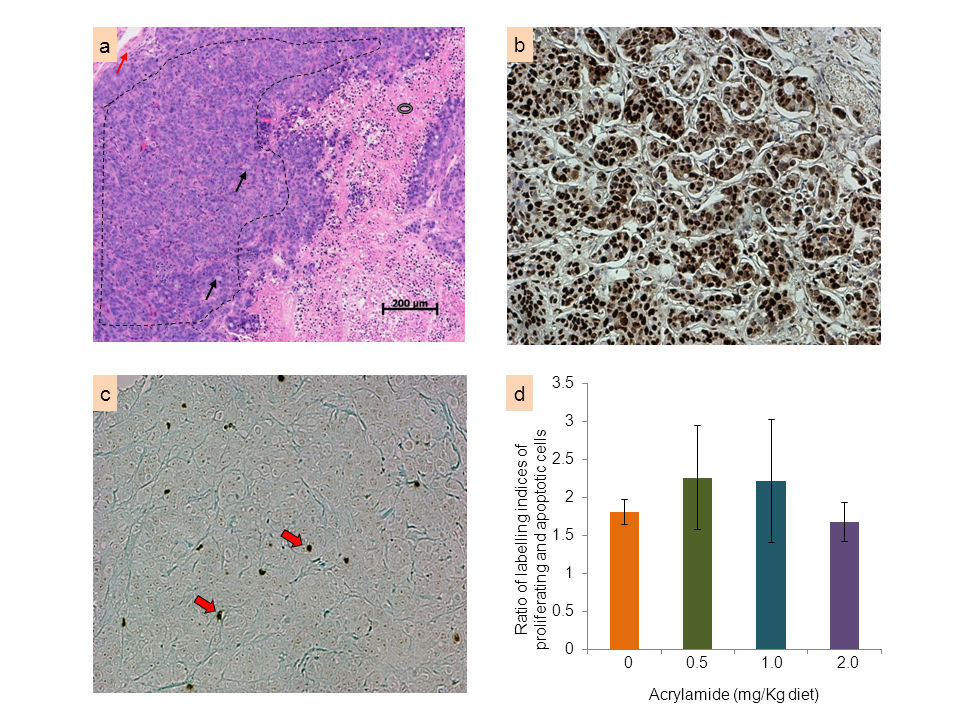
Histology, proliferation and apoptosis of tumor xenograft characteristics in nude (*nu/nu*) mice. Fixed HT-29 human colon tumor xenografts from nude (*nu/nu*) mice were sectioned at 5 µm. Panel (a) shows a representative H&E section of the tumor xenografts as a typical undifferentiated carcinoma with viable solid sheets of tumor cells (area marked by black dotted line) and clearly delineated subcutis margin (red arrow); key characteristics include grey distending tumor cell cytoplasm (black arrow) and the necrotic area (black donut). Representative immunohistochemical staining of PCNA is shown in panel (b) with proliferating cells seen as dark brown stained cells. TUNEL staining is shown in panel (c) with apoptotic cells observed as dark spots (red arrow). The bar graph in panel (d) shows the ratio of the indices (mean ± SE) of proliferating (PCNA-positive) and apoptotic (TUNEL-positive) cells for each diet: acrylamide at 0 (control), 0.5, 1.0 and 2.0 mg/kg diet.

H&E sections of all fixed tumor xenografts were assessed. The morphology of the tumors was very similar among the 4 dietary groups. Consistently, all tumors were clearly delineated, unencapsulated, densely cellular masses of tumor cells that were divided into variably sized lobules by a fine fibrovascular stroma (Figure 6). Large areas of necrosis within the masses were a constant feature, with the periphery of the tumors infiltrated by a mixed population of leukocytes. Under the microscope, at low objective, the tumors appeared solid and without glandular differentiation; at high objective, occasional to frequent small lumens occurred among the cancer cells. The cells had a high nucleus/cytoplasmic ratio, marked anisokaryosis (variation in diameter of nucleus), and densely stained chromatin with large single or multiple nucleoli. Mitotic cells, including atypical ones, were frequent. Multinucleated cells were a common feature. Grey mucus was present in the cytoplasm as either small inclusions or was so voluminous as to cause nuclear compression (signet-ring cells).

Immunohistochemistry analyses to determine mitotic (PCNA-labelled) and apoptotic cell counts were performed in unstained sections of the tumor xenografts (Figure 6). A ratio of the labeling indexes of mitotic and apoptotic cells were calculated for each tumor xenografts; there were no changes in this ratio between control and acrylamide-treated groups (Figure 6).

## Discussion

Acrylamide is known to cause tumors in multiple organs in rodent bioassays [1,2,4]. However, in these studies, the colon was not identified as a target organ for the carcinogenic effect of acrylamide. Two human epidemiological studies failed to observe any relationship between acrylamide intake and the risk of developing colon cancer [18,19], with a reiteration of this point by a recent meta-analysis study [27]. Contrary to these findings and those of the carcinogenicity experiments, a study conducted in Sprague-Dawley rats reported that i.p. exposure to acrylamide (10 mg/kg body weight 5 days a week for 8 weeks) induced colon ACF and tumor formation [20]. In addition, compared to rats on a basal diet, those that were fed diets supplemented with 10% corn oil and 10% beef tallow had more, and those fed diets supplemented with 10% olive oil and 10% fish oil had fewer acrylamide-induced colon ACF [21], using a dose of acrylamide similar to that reported earlier [20]. The studies of Zhang (2009) and Xichun (2009) suggest that acrylamide may be involved in a direct carcinogenic action in the colon and that the acrylamide-induced colon lesions are amenable to growth regulation by dietary lipids [20,21]. Earlier, we conducted an eight-week study in AOM-injected F344 rats fed diets with acrylamide at doses known to cause multiple tumors in rodents [1,2,4]; we reported that there was no increase in colon ACF formation, a surrogate biomarker of colon tumorigenesis [22]. In the present study, acrylamide was added in the diet to reflect the upper levels found in popular human foods, which was lower than those used in previous studies including our own study [1,2,4,22]. We used the well-established AOM-induced rat colon cancer model to understand if dietary acrylamide initiated colon tumors. AOM-induced rats develop colon tumors similar to those found in the human colon. This animal model is ideal to study colon tumorigenesis with an opportunity to intervene with chemicals, drugs or nutrients in a medium-term time frame (24-30 weeks post AOM-treatment), thus targeting a chronic and sustainable exposure [28,29]. Colon tumor data derived from studies using the AOM rat model have been successful in assessing preclinical efficacy relating to human relevance [29]. Additionally, we examined if HT-29 human colon cancer xenografts in nude (*nu/nu*) mice were amenable to tumor promotion by dietary acrylamide.

Our experimental strategy aimed to specifically assess the potency of acrylamide that is food-borne. The calculated intake of acrylamide based on the average food consumption of the highest dose of 2.0 mg acrylamide/kg diet was ~ 70 µg and 72 µg per kg body weight in saline- and AOM-injected F344 rats, respectively, and ~ 25 µg per kg body weight in nude (*nu/nu*) mice. In humans, the safe daily dietary intake level for acrylamide was estimated to range between 1–4 µg per kg body weight over a lifetime [30]. Thus, our dietary doses ranging from 17–70 and 6-25 µg acrylamide per kg body weight per day in F344 rats and nude (*nu/nu*) mice, respectively, are relevant for comparison to exposure observed in humans and amounts present in certain common foods [9–12]. Indeed, a careful consideration of species difference and nonlinear processes (including toxicokinetic and toxicodynamic differences) as suggested by Gargas et al. (2009) [31], in extrapolating our results for estimating risk and safety of acrylamide ingested by humans is warranted.

Under experimental conditions, in both the F344 rat and the nude (*nu/nu*) mouse studies, dietary acrylamide did not result in any treatment-related adverse effects on body weight, body weight gain, food consumption, organ weights and the general health of animals. In saline-injected F344 rats, acrylamide (at all three doses tested) did not induce the formation of visible colon tumors. Additionally, we microscopically scanned all the colons from the saline-injected rats (fixed flat for more than 24 hours in formalin and stained with 0.2% methylene blue) according to the method by Bird, 1987 [32]; we observed no microscopic lesions or ACF. These results suggest that food-borne acrylamide, at doses similar to those found in human foods, is unable to independently induce any carcinogenic action in the colon. Our results are strikingly different from that reported by Zhang (2009) and Xichun (2009) [20,21]. We attribute this inability of acrylamide to induce colon tumors to (a) the low dose, and (b) the route of administration we have used compared to that used by others [20,21]. In addition, the low level of acrylamide used in this study (~ <70 µg acrylamide per kg body weight per day) may not metabolize into its active epoxide metabolite glycidamide in amounts that could cause any carcinogenic action in the colon. It is well accepted that the carcinogenicity of acrylamide is dependent on its metabolism to glycidamide [33,34].

In the AOM-injected F344 rats, colon tumor incidences and multiplicities were comparable between the control (rats that received diets with no added acrylamide) and the acrylamide-treated groups. The exception to this was that a notably higher tumor incidence (with no variation in multiplicity) was observed in the highest acrylamide dose group compared to the control. Interestingly, only at the highest dose of 2.0 mg acrylamide per kg diet did we observe a significantly greater size in the colon tumors compared to the control. In addition, there were more colon adenocarcinomas than adenomas in the highest dose group (2.0 mg/kg acrylamide per kg diet) compared with controls (which had more adenomas than adenocarcinomas in the colon). These results suggest that acrylamide at the highest dose of 2.0 mg acrylamide per kg diet may act as a co-carcinogen in the presence of AOM, a known colon-specific carcinogen. In our previous study [22], we observed an increase in the number of colon ACF in the proximal colons of AOM-injected F344 rats treated with 5 mg/kg acrylamide/diet compared to control (no acrylamide); however, ACF were lower in the other dose groups of 10 mg/kg and 50 mg/kg acrylamide/diet [22]. In that study, the doses used were based on those known to cause rodent tumors in rodents from the 2-year carcinogenicity studies of acrylamide [4,5]. In the current study, we observed a total of 2/22 and 5/26 tumors in the proximal colon regions of the control and the highest dose (2 mg/kg) acrylamide/diet groups, respectively. The doses that induced changes in the colons from the previous and current studies (5 mg/kg acrylamide/diet augmenting ACF and 2 mg/kg acrylamide/diet increasing tumor size, respectively) are relatable, given the changes occurred in the proximal regions of the colon and both studies had the same AOM regimens. This suggests that the co-carcinogenic sequence of events may involve targets early on, in the precancerous stages. A similar co-carcinogenic effect of acrylamide was noted in *N*-methyl-*N*-nitrosourea (MNU)-induced Sprague-Dawley rats; the multiplicity of mammary gland tumors (adenocarcinomas) was significantly higher in rats treated with 40 ppm acrylamide (in drinking water) compared to controls (rats induced with MNU with no acrylamide treatment) [35]. In the same study, the authors reported that acrylamide failed to exacerbate mammary gland tumors initiated by 7,12-dimethylbenz(a) anthracene and promoted by *N*-bis(2-hydroxypropyl) nitrosoamine [35]. The carcinogen-dependence of acrylamide seems to involve an H-*ras* point mutation and infrequent chromosomal alterations commonly observed in MNU-induced carcinogenesis [35,36]. In a genetic model of intestinal tumorigenesis, acrylamide and glycidamide elicited tumorigenic activity in the small intestines of *Min*/+ mice and their wild-type counterparts, with the main tumorigenic effect observed with perinatal exposure to glycidamide rather than acrylamide [37]. In a similar experimental strategy, neonatal male B6C3F_1_ mice were given i.p. treatments (at postnatal days 1, 8 and 15) of acrylamide or glycidamide at two doses each of 0.14 or 0.70 mmol per kg body weight per day [34]. The authors reported a higher incidence of liver tumors in the 0.70 mmol per kg body weight glycidamide group compared to the control group, this was associated with A→G and A→T mutations at codon 61 of the H-*ras* oncogene in the liver tumors, suggesting a more crucial role of glycidamide than acrylamide in the carcinogenic action [34]. The co-carcinogenic effects of acrylamide in the colon (and possibly in other organs) and, more importantly, the effects of its active metabolite glycidamide needs more scrutiny, not only in relation to known chemical carcinogens (wherein carcinogen synergism is observed) but to other agents such as endocrine disruptors and pre-disposing genetic factors of carcinogenesis such as inflammation and obesity.

The nude (*nu/nu*) mice were used as a model to investigate the effects of food-borne acrylamide on established colon tumors of human origin. There were no changes in the pattern of growth in the palpable colon tumor xenografts between control and acrylamide-treated mice. These results are further strengthened by the fact that no changes were observed in the ratio of the indices of proliferating (PCNA-positive) cells and apoptotic (TUNEL-positive) cells between control and acrylamide-treated mice. One of the limitations in the results of the nude mice study is that the acrylamide effects cannot be directly compared to those from our saline/AOM-injected F344 rat study due to the fact that the acrylamide exposures calculated on the basis of food intake are lower in the mouse study. Nevertheless, the highest dose of 2.0 mg/kg diet amounts to an acrylamide intake of ~ 25 µg per kg body weight in nude (*nu/nu*) mice, based on their food consumption, and these values are comparable to those found in popular foods for human consumption and upper human exposure levels [9–12].

Here, we have assessed if dietary exposure of acrylamide, at levels relevant to those found in popular human foods, caused any tumorigenic effects in the colon using two established models. We conclude that acrylamide intake, at doses within the same order of magnitude as from the human diet, did not induce the formation of colon tumors in saline-treated rats, suggesting that it is not a complete carcinogen to the colon. The highest dose of acrylamide (2 mg/kg diet) increased the size of the tumors in AOM-injected rats and resulted in a higher occurrence of adenocarcinoma-type lesions, suggesting that acrylamide may act as a co-carcinogen in the colon and exacerbate the effects of AOM (a known colon-specific carcinogen). In the nude (*nu/nu*) mouse model, dietary acrylamide did not aid in the progression of established human colon tumor xenografts, and hence is not a tumor promoter in the colon by itself. The role food-borne acrylamide may play as a co-carcinogen in the colon (and in other organ sites), interacting with potential genetic (obesity/inflammation) or environmental (endocrine disruptors) tumor initiators, warrants further investigation. To understand the mechanism(s) of acrylamide-related tumor changes, we are currently analyzing the colon tissues (tumors and normal-appearing mucosae from F344 rats and the human colon tumor xenografts from nude mice) for genomic and epigenomic markers. These data provide government food regulators with information to better define the health risks and hazard characterization of dietary acrylamide and to develop an understanding of the relevance of such experimental evidence to humans.
