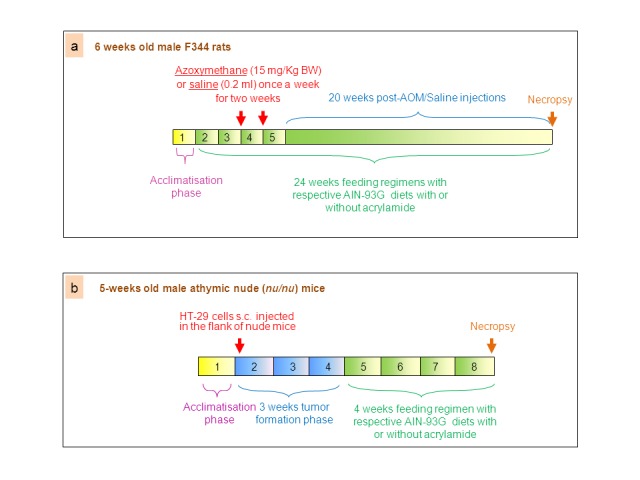# Correction: Negligible Colon Cancer Risk from Food-Borne Acrylamide Exposure in Male F344 Rats and Nude (*nu/nu*) Mice-Bearing Human Colon Tumor Xenografts

**DOI:** 10.1371/annotation/f040499f-8485-4f7f-88bd-6720379064e9

**Published:** 2013-09-11

**Authors:** Jayadev Raju, Jennifer Roberts, Chandni Sondagar, Kamla Kapal, Syed A. Aziz, Don Caldwell, Rekha Mehta

The version of Figure 1 that appears in the article is incorrect. The correct version is available here: 

**Figure:**